# The Temporal Dynamics of Loneliness and Cognitive Function in Older Adults

**DOI:** 10.1093/geroni/igaf122.1298

**Published:** 2025-12-31

**Authors:** Jay Kayser, Xiaoling Xiang, Jacqui Smith

**Affiliations:** University of Michigan, Ann Arbor, Michigan, United States; University of Michigan-Ann Arbor, Ann Arbor, Michigan, United States; University of Michigan, Ann Arbor, Michigan, United States

## Abstract

The life course stress model suggests that stressors, such as loneliness, can accumulate and shape long-term cognitive trajectories, particularly in later life. While loneliness has been linked to cognitive decline, few studies have examined whether this relationship is bidirectional. This study investigated associations between loneliness and cognitive function over time. Data were drawn from four waves (2008–2022) of the Health and Retirement Study. The analytic sample included 12,144 adults aged 50 and older. A random-intercept cross-lagged panel model was used to separate within-person fluctuations from stable between-person differences, and a dual-process latent growth curve model examined long-term patterns in loneliness and cognitive function. Loneliness was measured using the 11-item UCLA Loneliness Scale, and cognition was assessed via immediate and delayed word recall tasks. Cross-lagged effects pathways between loneliness and cognitive function were not statistically significant, suggesting no within-person bidirectional relationship. However, the random intercepts for cognition and loneliness were negatively correlated (r = -0.22, p < .001), suggesting that individuals with higher loneliness tended to have lower cognitive function at baseline. Additionally, the random slopes for cognition and loneliness were also negatively correlated (r = -0.30, p < .001), indicating that individuals with greater declines in cognition also tended to experience increases in loneliness over time. These findings suggest that loneliness and cognitive function are interconnected, even in the absence of short-term directional effects. Understanding their long-term association can inform interventions aimed at preserving cognitive health and reducing loneliness in older adults.

